# A case report of severe systemic herpes simplex virus-1 (HSV-1) infection with multi-organ involvement after a course of oral corticosteroid treatment

**DOI:** 10.1186/s12879-022-07815-3

**Published:** 2022-11-05

**Authors:** Myeongji Kim, Ayesha Jalal, Heysu Rubio-Gomez, Romina Bromberg

**Affiliations:** 1 Department of Internal Medicine, Memorial Healthcare System, 703 N. Flamingo Rd, GME 2nd Floor, 33028 Pembroke Pines, FL USA; 2 Division of Infectious Disease, Memorial Healthcare System, 5647 Hollywood Blvd, 33021 Hollywood, FL USA

**Keywords:** Herpes simplex virus, Disseminated infection, Multi-organ failure, Acyclovir, Case report

## Abstract

**Background:**

Herpes simplex virus (HSV) rarely causes organ-invasive infection. Diagnosis and treatment for such infections are often delayed, and mortality is high. We present the first reported case of disseminated HSV-1 infection in an adult causing liver failure, myocarditis, and encephalitis in a patient who recovered after receiving parenteral acyclovir treatment.

**Case presentation:**

A 46-year-old female presented with fever, chills, and malaise after 2 weeks of oral corticosteroid treatment for uveitis. She was diagnosed with disseminated HSV-1 infection with multi-organ involvement causing hepatitis, encephalitis, and myocarditis. Diagnosis was made timely using serum polymerase chain reaction (PCR) for HSV DNA and the patient was given intravenous acyclovir treatment promptly, which led to her survival without significant morbidity.

**Conclusions:**

Clinicians should have a low threshold for suspecting HSV infection and ordering HSV PCR to decrease morbidity and mortality when there is a high clinical suspicion of systemic HSV infection with multi-organ involvement. Serum PCR for HSV DNA is an excellent modality for an initial diagnostic approach. Further research is warranted to elucidate causality between a course of corticosteroid therapy and systemic HSV-1 infection without major immunosuppressive comorbidities or treatments.

## Background

Herpes simplex virus (HSV), a member of *Herpesviridae* family, is an enveloped, double-stranded DNA virus commonly causing dormant infection of neural ganglia [1]. However, HSV can also cause life-threatening infections such as hepatitis, encephalitis, and myocarditis. Other manifestations of HSV infection include vesicular rash, esophagitis, keratitis, and pneumonia [2–6]. Although hepatitis caused by HSV infection is rare, accounting for about 1% of cases of acute liver failure (ALF) in adults [7], it can be fatal if left untreated. Norvell et al., in 2007, found an overall 74% mortality for all cases of HSV hepatitis reported since 1969 [7]. Unfortunately, even for the patients treated with acyclovir, mortality remains approximately 50% [7]. The other organ-invasive infection caused by HSV, herpes simplex encephalitis (HSE), occurs in two to four cases per million per year, and may lead to grim complications such as central nervous system vasculitis and autoimmune encephalitis [8]. Myocarditis caused by HSV is even rarer, as prevalence is estimated to be less than 1% of acute myocarditis [9]. Only one case of HSV-induced cardiomyopathy that was successfully treated has been reported [10]. Systemic disease with multi-organ involvement (with or without mucocutaneous lesions) is rarely reported. To our knowledge, this is the first reported case of disseminated HSV-1 infection in an adult with multi-organ involvement, causing liver failure, myocarditis, and encephalitis, with remarkable recovery of the patient after receiving parenteral acyclovir treatment.

## Case presentation

A 46-year-old female with medical history significant for class II obesity and left eye uveitis presented to the emergency department of a tertiary hospital in November 2021 complaining of non-resolving fever, chills, and malaise while undergoing treatment for a urinary tract infection. She also had an extensive ophthalmologic history; the patient was diagnosed with idiopathic multifocal choroiditis and uveitic glaucoma of the left eye in 2016. She underwent treatment for glaucoma with an implant and subsequently developed retinal scarring in the left eye. She was on adalimumab for two years until January 2020. When she presented to the emergency department, she had been taking prednisone 60 mg daily for the past two weeks due to acute exacerbation of uveitis. From January 2020 to acute exacerbation this time, her uveitis was in remission and she did not receive recurrent courses of corticosteroid treatment. At the time of presentation, she was not taking other medications or supplements except trimethoprim-sulfamethoxazole for a urinary tract infection, prednisone, and ibuprofen as needed.

**Fig. 1:**
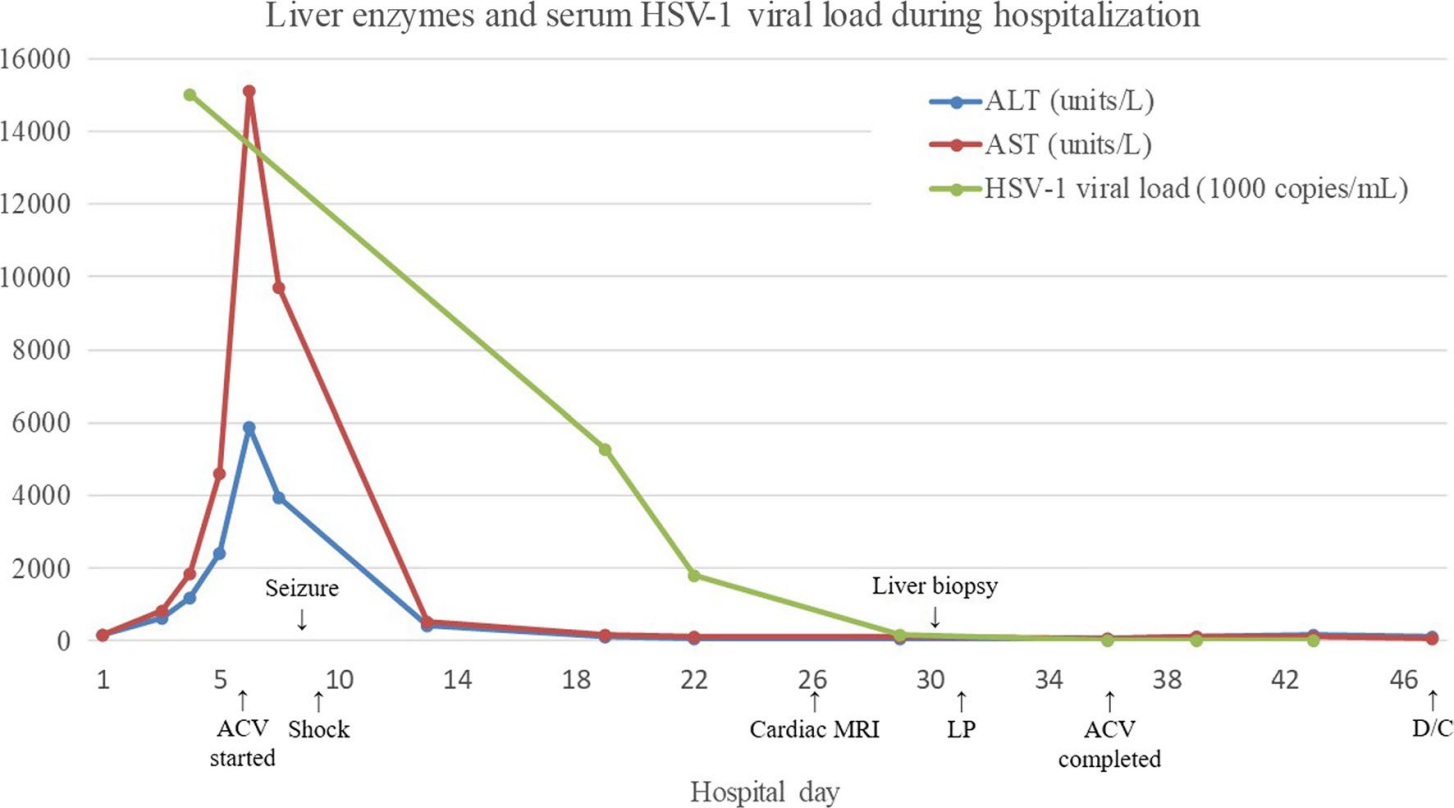
Liver enzymes, and serum HSV-1 viral load during hospitalization. *HSV-1* herpes simplex virus-1, *ALT* alanine transaminase, *AST* aspartate transaminase, *ACV* acyclovir, *MRI* magnetic resonance imaging, *LP* lumbar puncture, *D/C* discharge

Initial vital signs were blood pressure of 89 mmHg/74 mmHg, pulse rate of 117 beats/minute, respiratory rate of 26/minute, oral temperature of 39.7 ºC, and oxygen saturation of 96% on ambient air. Computed tomography (CT) imaging of abdomen and pelvis without intravenous (IV) contrast reported no acute findings identified in the abdomen or pelvis. Laboratory studies were significant for alanine transaminase (ALT) of 147 units/L and aspartate transaminase (AST) of 196 units/L. Urinalysis revealed trace ketones, 6–10 white blood cells/HPF, 6–10 red blood cells/HPF, and rare bacteria. Urine culture showed < 10,000 CFU/mL of multiple organisms. A set of blood cultures showed no growth.

The patient was empirically started on cefepime IV 1 g every 12 h. However, the patient had persistent fever along with worsening liver tests with hepatocellular pattern (ALT 608 units/L, AST 836 units/L, alkaline phosphatase (ALP) 94 units/L, total bilirubin 0.5 mg/dL) (Fig. 1). On hospital day 3, diagnostic tests for infectious etiology of acute hepatitis were ordered, including serology and quantitative polymerase chain reaction (PCR) for HSV and cytomegalovirus (CMV), syphilis serology, and interferon gamma release assay for tuberculosis. During hospital day 3 to day 6, the patient had persistent fever and increasing liver enzymes while undergoing treatment with IV cefepime (Fig. 1). On hospital day 6, serum HSV-1 PCR resulted positive with viral load of 1.50 × 10^7^ copies/mL. IV acyclovir was started at the dose of 10 mg/kg every 8 h and IV cefepime was discontinued. Serum HSV-1 immunoglobulin M (IgM) titer was elevated to greater than 1:320 and HSV-1 IgG titer was lower than threshold of detection. Other work-up for infectious etiologies all resulted negative. Non-infectious etiologies for acute hepatitis were explored; plasma acetaminophen level, total immunoglobulin A (IgA), total immunoglobulin G (IgG), actin-smooth muscle antibody, antineutrophil cytoplasmic antibody (ANCA) screening, soluble liver antigen autoantibody, tissue transglutaminase IgG, M2 mitochondrial antibody, liver/kidney microsomal antibody, ceruloplasmin were all unremarkable.

On hospital day 7, liver enzymes peaked at ALT of 5885 units/L and AST of 15,107 units/L. The patient’s mental status changed, with new-onset confusion and agitation. With acute deterioration in mental status along with worsening liver failure, patient required admission to the intensive care unit (ICU). The next day, the patient had a witnessed tonic-clonic seizure for 30 s and was emergently intubated for airway protection. CT of the brain was negative for an acute intracranial process. On hospital day 9, the patient became hypotensive requiring vasopressor support with norepinephrine infusion. Serum troponin I was elevated to 18.38 ng/mL. Transesophageal echocardiogram revealed severe left ventricular hypokinesia with an approximate ejection fraction of 10%. The patient also had episodes of paroxysmal non-sustained ventricular tachycardia. At this point, the patient was evaluated for liver transplantation, however, was deemed not a transplant candidate due to severe cardiomyopathy and down-trending liver transaminases after starting IV acyclovir treatment. On hospital day 18, the patient was successfully extubated, and subsequently noted to be encephalopathic with disorientation and hallucination. On hospital day 26, cardiac magnetic resonance imaging (MRI) revealed left ventricular ejection fraction recovered to 43%, along with myocardial scar and thinning involving mid-apical inferolateral segment of left ventricle, with corresponding hypokinesis involving mid-inferolateral, antero-lateral, and apico-lateral segments. On hospital day 30, transjugular liver biopsy was performed and pathology showed acute hepatitis with sub-massive necrosis, with negative HSV-1 and HSV-2 antigen immunostaining. On hospital day 31, lumbar puncture was performed, and cerebrospinal fluid showed white blood cell count, protein, and glucose, all within normal parameters, however a positive PCR for HSV-1. During hospital day 33 to 36, patient developed acute worsening of thrombocytopenia, as platelet count decreased from 107,000/µL to 2000/µL. On hospital day 36, acyclovir treatment was held for one day, with improvement of platelet count to 32,000/µL. On the same day, serum HSV-1 PCR showed viral load of 7700 copies/mL. The patient’s mental status, liver function tests and coagulation panel all continued to improve. Given the patient’s clinical progress, decreasing viral load, and improvement in thrombocytopenia with cessation of acyclovir treatment, the decision was made to complete acyclovir treatment at that point. On hospital day 43, HSV-1 serum viral load further decreased to 2488 copies/mL without acyclovir treatment. The patient was discharged to a rehabilitation facility on hospital day 47 in a stable condition. After a month of comprehensive rehabilitation the patient was successfully discharged home.

## Discussion and conclusion

### Immune status and disseminated HSV infection

Systemic HSV infection is more common in immunocompromised individuals, such as transplant recipients, patients receiving long-term corticosteroid therapy, and individuals with burn-related injuries. Pregnant women and neonates also account for a large number of cases. Immunocompetent, non-pregnant adults account for 21–24% of all patients with HSV hepatitis in prior case reports [7]. Of note, we have found two case reports where an immunocompetent patient developed fatal HSV hepatitis, after a brief course of systemic corticosteroid therapy for other indications such as asthma exacerbation or brain edema [11, 12]. Efforts to understand the immunosuppressive effect of glucocorticoid is still ongoing [13]. Most of the immunosuppressive properties of glucocorticoids are thought to occur through the glucocorticoid receptor; once activated in leukocytes, glucocorticoid receptor alters transcription of numerous genes involved in acute and chronic inflammation [14]. These genes encode for pro-inflammatory cytokines, cell adhesion molecules, and key enzymes in initiation and maintenance of host inflammatory response [14]. As described above, a brief course of corticosteroids for uveitis was the risk factor identified in our patient that led her to develop fulminant multi-organ HSV infection in an otherwise healthy female. A course of systemic steroid therapy is utilized quite often as part of therapeutic plan for various medical conditions. The morbidity and mortality of fulminant HSV stresses the need for further research on systemic corticosteroids as a potential risk factor for developing this disease manifestation.

### Establishing the diagnosis of organ-invasive HSV infection

Gold standard diagnostic tests for HSV hepatitis, encephalitis, and myocarditis involve invasive modalities. PCR CSF analysis is considered the gold standard in diagnosis of HSE, and in our case CSF was positive for HSV-1 PCR, well establishing the diagnosis. On the other hand, diagnosis of HSV hepatitis and myocarditis in this case is more presumptive, as liver biopsy was negative for HSV-1 immunostaining and endomyocardial biopsy was not performed. The diagnosis of HSV-1 hepatitis is strongly supported by the fact that extensive infectious and noninfectious work-up for hepatitis were negative except for positive HSV-1 PCR and IgM. In addition, the patient showed clinical improvement with IV acyclovir treatment. When liver biopsy was performed, the patient had already received 24 days of acyclovir treatment. Therefore, the liver biopsy result of acute hepatitis with sub-massive necrosis, with negative HSV-1 viral stain does not exclude HSV-1 as an etiology of hepatitis in this patient. Regarding the diagnosis of HSV myocarditis, evaluation was limited as full cardiac work-up was not completed including cardiac catheterization. Cardiac MRI cardiac showed recovered ejection fraction and myocardial scarring, thinning, and hypokinesis involving the mid-apical inferolateral segment of the left ventricle. These finding are nonspecific and both viral myocarditis and ischemic insult might have led to these changes. In the setting of incomplete work-up, the diagnosis of HSV myocarditis is not definitive, however, still highly suggestive in the setting of severe viremia and systemic disease.

### Early diagnosis and treatment

Of the 52 HSV hepatitis cases reviewed by Kaufman et al., the diagnosis was made in the antemortem period for only 23% of the cases [15]. Due to this limitation, a 1997 review of patients with HSV hepatitis noted that only 28% of patients received antiviral agents, yet by 2007, this rate had increased to only 37% [7, 11]. On the other hand, serum PCR assay of HSV DNA is relatively fast and noninvasive, and has a sensitivity and specificity of > 95% for HSV viremia, variability dependent on assay and laboratory. In a study using stored sera obtained from patients enrolled in the United States ALF Study Group database, all four known HSV cases had high-titer HSV DNA on presentation [16]. Given the limitations of performing invasive organ specific gold standard tests, PCR may turn out to be a lifesaving approach. In addition, early suspicion of infection is imperative in both immunocompetent and immunocompromised patients, as a delayed diagnosis leads to high mortality [7]. Disseminated HSV infection often lacks specific symptoms and should be considered in the setting of absence of mucocutaneous lesions with findings of either highly elevated liver enzymes, coagulopathy, altered mental status, or rapid progression of cardiomyopathy [7]. Importance of obtaining HSV serum PCR as early as possible can be further emphasized, as viral PCR may take longer in non-tertiary institutions and time to initiation of acyclovir treatment is paramount. Acyclovir, a nucleoside analogue which inhibits viral DNA polymerase activity, is the treatment of choice for disseminated HSV. Foscarnet is an alternative therapy, and the efficacy of cidofovir is currently uncertain in that context. In a study, clinical samples of 12 HSE patients were evaluated and intrathecal resistance of acyclovir was not detected [17]. Several studies have found that in disseminated HSV infection, longer time from symptom onset to IV acyclovir initiation was associated with higher mortality [7, 18]. In our case, HSV serum PCR was ordered immediately, which led to a prompt diagnosis and treatment with parenteral acyclovir, ultimately resulting in the patient’s survival.

We described a case of systemic HSV-1 infection involving multiple organ systems leading to acute liver failure, cardiomyopathy, and encephalitis, after the patient received 2-week course of oral corticosteroid treatment. The patient recovered successfully without major morbidity with parenteral acyclovir treatment. We suggest a course of corticosteroid therapy can precipitate systemic HSV-1 infection even without a major immunosuppressive comorbidity or treatment. Also, manifestations of systemic HSV-1 infection is nonspecific; thus, clinicians should have a low threshold for suspecting HSV-1 infection in the setting of multi-organ failure without a clear etiology. We also suggest that serum PCR for HSV DNA is an excellent modality for an initial diagnostic approach; it is noninvasive, readily-available, and has a high sensitivity and specificity.

## Data Availability

Data sharing is not applicable to this article as no datasets were generated or analyzed during the current study.

## Abbreviations

ALF: Acute liver failure
ALP: Alkaline phophatase
ALT: Alanine transaminase
ANCA: Antineutrophil cytoplasmic antibody
AST: Aspartate transaminase
CMV: Cytomegalovirus
CT: Computed tomography
HSE: Herpes simplex encephalitis
HSV: Herpes simplex virus
ICU: Intensive care unit
IgA: Immunoglobulin A
IgG: Immunoglobulin G
IgM: Immunoglobulin M
IV: Intravenous
MRI: Magnetic resonance imaging
PCR: Polymerase chain reaction
